# Right coronary wall cmr in the older asymptomatic advance cohort: positive remodeling and associations with type 2 diabetes and coronary calcium

**DOI:** 10.1186/1532-429X-12-75

**Published:** 2010-12-30

**Authors:** Masahiro Terashima, Patricia K Nguyen, Geoffrey D Rubin, Craig H Meyer, Ann Shimakawa, Dwight G Nishimura, Shoichi Ehara, Carlos Iribarren, Brian K Courtney, Alan S Go, Mark A Hlatky, Stephen P Fortmann, Michael V McConnell

**Affiliations:** 1 Division of Cardiovascular Medicine, Stanford University School of Medicine, Stanford, CA, USA; 2 Department of Radiology, Stanford University School of Medicine, Stanford, CA, USA; 3 Department of Biomedical Engineering, University of Virginia, Charlottesville, VA, USA; 4 Applied Science Laboratory-West, GE Healthcare, Menlo Park, CA, USA; 5 Magnetic Resonance Systems Research Laboratory, Department of Electrical Engineering, Stanford University School of Medicine, Stanford, CA, USA; 6 Division of Research, Kaiser Permanente of Northern California, Oakland, CA, USA; 7 Departments of Epidemiology, Biostatistics, and Medicine, University of California, San Francisco, San Francisco, CA, USA; 8 Department of Health Research and Policy, Stanford University School of Medicine, Stanford, CA, USA; 9 Stanford Prevention Research Center, Stanford University School of Medicine, Stanford, CA, USA

## Abstract

**Background:**

Coronary wall cardiovascular magnetic resonance (CMR) is a promising noninvasive approach to assess subclinical atherosclerosis, but data are limited in subjects over 60 years old, who are at increased risk. The purpose of the study was to evaluate coronary wall CMR in an asymptomatic older cohort.

**Results:**

Cross-sectional images of the proximal right coronary artery (RCA) were acquired using spiral black-blood coronary CMR (0.7 mm resolution) in 223 older, community-based patients without a history of cardiovascular disease (age 60-72 years old, 38% female). Coronary measurements (total vessel area, lumen area, wall area, and wall thickness) had small intra- and inter-observer variabilities (r = 0.93~0.99, all p < 0.0001), though one-third of these older subjects had suboptimal image quality. Increased coronary wall thickness correlated with increased coronary vessel area (p < 0.0001), consistent with positive remodeling. On multivariate analysis, type 2 diabetes was the only risk factor associated with increased coronary wall area and thickness (p = 0.03 and p = 0.007, respectively). Coronary wall CMR measures were also associated with coronary calcification (p = 0.01-0.03).

**Conclusions:**

Right coronary wall CMR in asymptomatic older subjects showed increased coronary atherosclerosis in subjects with type 2 diabetes as well as coronary calcification. Coronary wall CMR may contribute to the noninvasive assessment of subclinical coronary atherosclerosis in older, at-risk patient groups.

## Introduction

Noninvasive imaging techniques to assess subclinical coronary atherosclerosis have the potential to identify patients at higher risk for future coronary events and guide lifestyle modifications and pharmacological therapy to maximize cardiovascular risk reduction. Over the last decade, significant progress has been made in the development of coronary magnetic resonance angiography using a variety of techniques[1-5]. However, the assessment of coronary artery luminal narrowing has limited value for the detection of subclinical coronary atherosclerosis because the coronary artery lumen size is often preserved by positive arterial remodeling[6]. Therefore, direct noninvasive imaging of the coronary artery wall is an important target for cardiovascular imaging.

While coronary wall imaging by cardiovascular magnetic resonance (CMR) is challenging due to the small size of coronary arteries and cardiac/respiratory motion, it has been successfully applied in patients using breathhold[7-9] or respiratory gating (i.e., free-breathing) techniques[10-14]. Using these techniques, significant increases in coronary wall area and wall thickness have been found in patients with documented coronary artery disease[10,12] and in those with type 1 diabetes mellitus plus nephropathy [13]. The MESA study more recently showed positive arterial remodeling by coronary wall CMR in 179 asymptomatic participants[14]. There are limited data on evaluating subclinical atherosclerosis in older patient groups, who are at increased risk for coronary events.

We have investigated clinical, imaging, and genetic abnormalities in an at-risk (i.e., older) community-based patient cohort - the Atherosclerotic Disease, Vascular Function, and Genetic Epidemiology (ADVANCE) study[15,16]. Here we report on coronary wall CMR in this older, asymptomatic cohort to assess image quality, measurement variability, remodeling, and risk factor associations.

## Methods

### Study Patients

This was an CMR sub-study of 223 participants from the ADVANCE study of 1023 older subjects (age 60 to 72 years, 34% women) with details on the patient cohort published previously[5,16]. Briefly, subjects without a known history of cardiovascular disease or other major comorbidities were randomly selected from the membership of Kaiser Permanente of Northern California. All subjects were studied through the Stanford Prevention Research Center. Coronary risk factors were measured using standardized protocols[17]. Diabetes mellitus was defined as self-report of physician-diagnosed diabetes, use of diabetes medication, or fasting blood glucose ≥ 126 mg/dl at the study visit[18]. Hypertension was defined as use of antihypertensive medication or blood pressure ≥ 140/90 mmHg at study visit. Multi-detector row spiral computed tomography (CT) was used to quantify coronary artery calcification (CAC) by the Agatston score, as previously reported[16,19].

Written informed consent was obtained from all participants. The study protocol was approved by the Institutional Review Boards of Stanford University and the Kaiser Foundation Research Institute.

### Coronary Wall Imaging by CMR

A 1.5T Signa MR scanner (GE Healthcare, Waukesha, WI) was used, equipped with high-performance gradients (40 mT/m, 150 mT/m/ms) and a commercial 4-channel cardiac phased-array surface coil (GE Healthcare, Waukesha, WI). A real-time interactive MR system (iDrive, GE Healthcare, Waukesha, WI) was used for coronary localization. For determination of the most quiescent period of the right coronary artery (RCA) within the cardiac cycle, a 2 D cine scan was performed in the 4-chamber view. High-resolution bright-blood coronary MRA, as previously reported[1,20], was performed to obtain in-plane views of the right coronary artery (RCA). Then, 3 cross-sectional coronary wall images of a linear portion of the proximal-to-mid RCA were acquired with 3 separate breathholds using a spiral black-blood coronary CMR sequence, with cardiac gating and acquisition during the previously identified patient-specific quiescent period. This sequence used an interleaved spiral k-space trajectory technique incorporating 1) double-inversion preparation (DIR) to null the blood signal, 2) spectral-spatial excitation for water-selective imaging, and 3) spiral readout (field of view = 22 cm, in-plane spatial resolution = 0.7 mm, slice thickness = 5 mm, slice gap = 0 mm, DIR thickness = 10 mm, TR = 1 heart beat, TE = 2.5 ms, TI = 280-350 ms adjusted for heart rate, flip angle = 90°, 18 interleaves, temporal acquisition window = 34 ms, scan time typically 20 sec per breathhold)[9].

### MR Image Analysis

**Figure 1:**
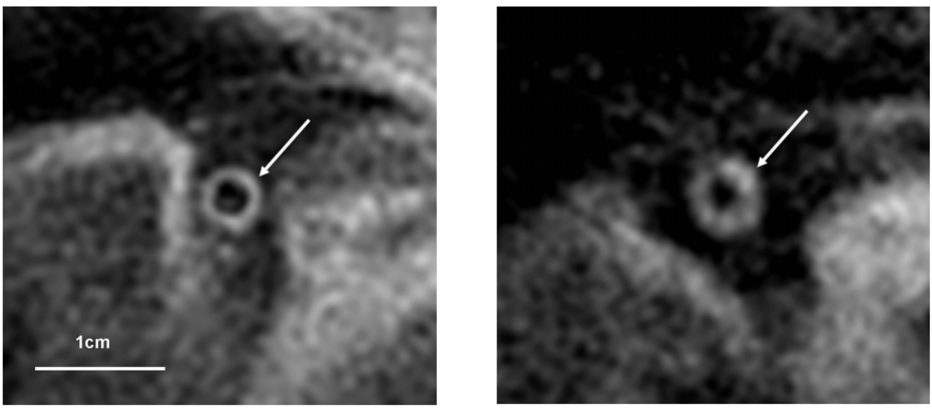
**Representative images of spiral black-blood coronary wall CMR (arrows) showing patients at the low and high end for mean coronary wall thickness (left - 1.27 mm, right - 2.06 mm)**.

Using the 3 cross-sectional RCA wall images, the image where the RCA wall exhibited the best image quality and the most circular cross-section was identified. Before quantitative analysis, image quality was rated on the MR images using a 4-point scale (4 = good, 3 = fair, 2 = poor, and 1 = very poor) based on signal-to-noise ratio (SNR), vessel border sharpness, and artifacts[21]. An image quality of 3 or above was considered adequate for quantitative analysis (Figure 1). These images were all pooled and then analyzed, blinded to patient information. Cross-sectional measurements of the total vessel area (VA) and lumen area (LA) of the RCA were performed manually using a custom-designed CMR analysis software after the images were magnified two-fold[5,22]. The coronary wall area (WA) was calculated as WA = VA-LA. The mean wall thickness (WT) was calculated assuming a circular vessel shape: WT = (√VA-√LA)/√π. All MR images were re-measured to assess intra- and inter-observer variability.

### Statistical Analysis

Continuous variables are expressed as means with standard deviations or medians with inter-quartile ranges. Differences between two groups for continuous variables were compared using Student's t-test or the Mann-Whitney U test. Differences in categorical variables among groups of interest were assessed using chi-squared tests. Measurements variability was assessed by both linear regression analysis and Bland-Altman analysis. Multivariate regression analysis was performed to identify predictors for increased coronary wall area and wall thickness. Traditional coronary risk factors, such as age, gender, body mass index (BMI), diabetes mellitus, hypertension, total/HDL cholesterol ratio, and current smoking were included in the final multivariate regression model. Spearman rank correlation analysis was performed for the relationship between CAC (log [CAC+1]) and coronary vessel area, lumen area, wall area, and wall thickness. A two-sided p value < 0.05 was considered statistically significant and all analyses were performed using StatView statistical software (SAS institute, Cary, North Carolina).

## Results

### Subject Characteristics

The mean age was 66 ± 3 years, 33% were women, and 37% were non-Caucasian (Table 1). Hypertension was common (56%), while 17% had type 2 diabetes mellitus and <8% were cigarette smokers. CAC > 0 was present in 78% (n = 173) of subjects.

**Table 1:** Clinical Characteristics of Study Subjects (n = 223)

Characteristic	
Age (years)	66.0 ± 2.7
Gender (men/women)	149/74
Body mass index (kg/m^2^)	27.0 ± 4.3
Diabetes mellitus (n, %)	38 (17)
Hypertension (n, %)	124 (56)
Total/HDL cholesterol (mg/dL)	3.7 ± 1.0
Current smoker (n, %)	17 (7.6)
Coronary artery calcium Agatston score (median, inter-quartile range)	39.5 (0.8-250.6)

### Coronary Wall Image Quality, Measurement Variability, Arterial Remodeling

**Figure 2:**
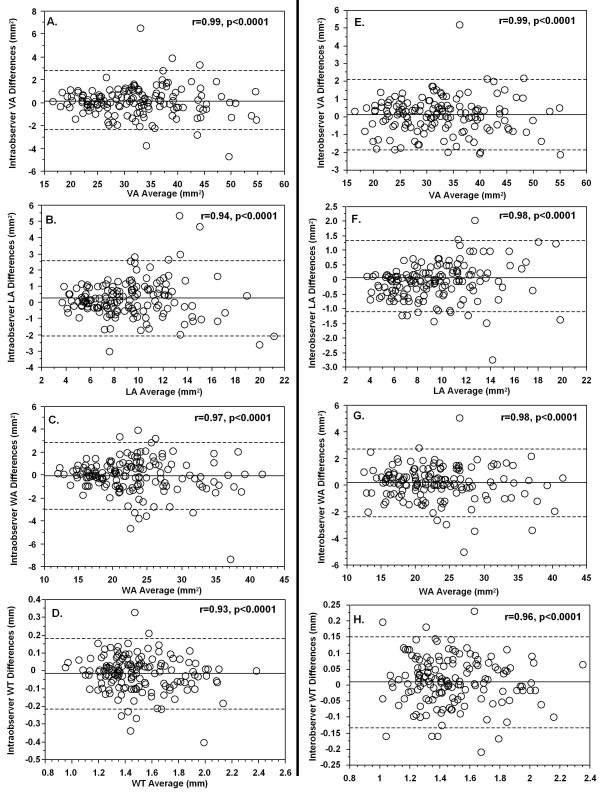
**Bland-Altman plots of intra-observer (left panels) and inter-observer (right panels) differences in coronary CMR measurements**. The solid lines are the mean differences and the dashed lines are 2 standard deviations (SD) from the mean difference. The linear regression analyses are also provided, with correlation coefficient and p value for each comparison.

Image quality of coronary wall CMR was >2 (fair or good) in 67% of subjects, leaving a total of 150 subjects for quantitative analysis. Bland-Altman analysis showed small differences for the intra- and inter-observer measurements of VA, WA, LA, and WT (Figure 2). The intra- and inter-observer variabilities were also low by linear regression analysis, with correlation coefficients of 0.93-0.99 (all p < 0.0001).

**Figure 3:**
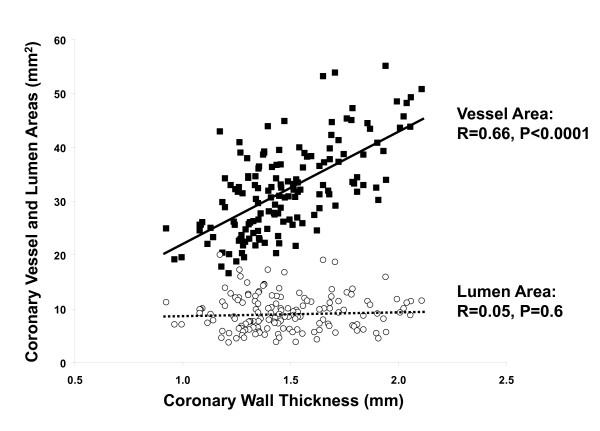
**Coronary wall thickness compared to both vessel area (solid squares, solid line) and lumen area (open circles, dashed line) by linear regression, showing significant positive correlation for vessel area but no significant change in lumen area, consistent with positive remodeling**.

Mean coronary measurements by CMR were as follows: VA = 32.1 ± 8.2 mm^2^, WA = 22.8 ± 5.9 mm^2^, LA = 9.3 ± 3.4 mm^2^, and WT = 1.48 ± 0.25 mm. VA increased with increasing WT (r = 0.66, p < 0.0001, Figure 3), while LA remained constant (r = 0.05, p = 0.6). The increase in VA, but not LA, with an increase in WT is consistent with positive arterial remodeling [10,14].

### Subclinical Coronary Atherosclerosis by CMR and Coronary Risk Factors

We compared coronary CMR measures to the clinical risk factors listed in Table 1. By univariate analysis, increased coronary WA and WT were associated with male gender (WA: 23.6 ± 5.9 vs. 21.7 ± 6.0 mm^2^, p = 0.045; WT: 1.51 ± 0.24 vs. 1.43 ± 0.26 mm, p = 0.046), diabetes (WA: 25.5 ± 6.2 vs. 22.4 ± 5.8 mm^2^, p = 0.04; WT: 1.61 ± 0.25 vs. 1.46 ± 0.24 mm, p = 0.008), and BMI (WA: r = 0.16, p = 0.046; WT: r = 0.2, p = 0.01). Total/HDL cholesterol ratio was associated with WT (r = 0.22, p = 0.008), but not WA. There were no significant associations between these risk factors and either VA or LA.

**Figure 4:**
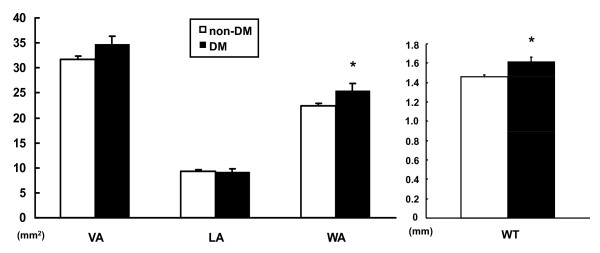
**Coronary wall CMR measurements between non-diabetic (n = 128) and diabetic (n = 22) subjects**. Both coronary wall area and wall thickness were significantly associated with diabetes mellitus (DM) by multivariate analysis. [* p < 0.05].

In multivariate analysis (Table 2), gender and BMI were no longer significant. Total/HDL cholesterol ratio remained independently associated with WT (p = 0.03), but not WA. Only diabetes was independently associated with both coronary WA (p = 0.04) and WT (p = 0.007). Differences in coronary measures between diabetics and non-diabetics are shown in Figure 4.

**Table 2:** Multivariate Regression Analysis of Coronary Risk Factors and Coronary Wall CMR

	Coronary WA (p = 0.038)			Coronary WT (p = 0.0009)		
**Variable**	**β value**	**SE**	**P Value**	**β value**	**SE**	**P Value**

Age (years)	-0.024	0.176	0.981	0.022	0.007	0.783
Gender (men/women)	0.099	1.033	0.239	0.101	0.042	0.216
Body mass index (kg/m^2^)	0.132	0.141	0.123	0.16	0.006	0.054
Diabetes mellitus (%)	0.177	1.402	***0.037***	0.223	0.057	***0.007***
Hypertension (%)	0.019	1.037	0.829	0.015	0.042	0.857
Total/HDL cholesterol (mg/dL)	0.105	0.49	0.226	0.189	0.020	***0.025***
Current smoker, n (%)	0.132	2.037	0.112	0.144	0.082	0.073

β: standardized regression coefficients. SE: standard error.

### Coronary Wall Measurements by CMR and Coronary Artery Calcification

**Figure 5:**
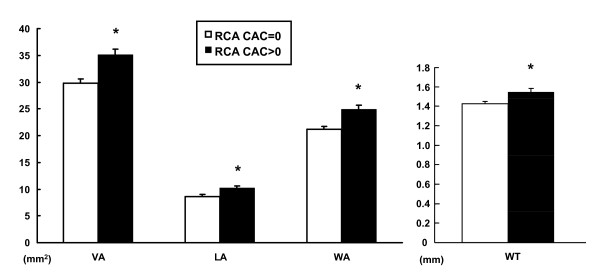
**Coronary wall CMR measurements and RCA calcification**. All coronary measurements were significantly greater in patients with positive RCA calcification (RCA CAC > 0, n = 84) compared to those without (RCA CAC = 0, n = 66). [* p < 0.005].

By Spearman rank correlation analysis, there was a significant correlation of CAC with coronary VA (p = 0.01), WA (p = 0.01), and WT (p = 0.03) (r = 0.18~0.21), but not coronary LA (p = 0.07). All coronary measurements were significantly increased in patients with any calcification of the RCA (p < 0.005, Figure 5).

## Discussion

We investigated coronary wall CMR in a cohort of asymptomatic older subjects, which to our knowledge is the largest group studied by coronary wall CMR to date and the first to focus on patients > 60 years old. The main findings were: 1) adequate coronary wall image quality was achieved in two thirds of the subjects and measurements had very good intra- and inter-observer variability, 2) coronary wall measurements were consistent with positive arterial remodeling, 3) coronary atherosclerosis by CMR was increased in subjects with type 2 diabetes and also 4) with increasing coronary calcification.

While coronary wall image quality and measurement variability have been reported in prior smaller studies, our study extends these findings to a large older community-based cohort where image quality can be challenging. This study confirms the positive arterial remodeling and association with coronary calcium reported in the MESA study, but now also in a large cohort of subjects over 60 years old. The association with type 2 diabetes is novel.

### Coronary Wall Imaging by CMR

Coronary wall CMR can be performed by breath-held and free breathing methods. To date, it has been shown to be reproducible in healthy volunteers[23,24] and to detect increased wall thickness in selected patients with known coronary artery disease, type 1 diabetes complicated by diabetic nephropathy, and asymptomatic individuals from the MESA study[10,12-14,25].

In our study, the breath-held spiral black-blood coronary CMR sequence provided adequate image quality in 67% of subjects (age 60-72 years). This was somewhat lower than the 74% of coronary locations visualized that had adequate image quality in the MESA study (where the age ranged from 45 to 84 years[25]). There are a number of factors that may contribute to inadequate image quality of coronary wall CMR, including suboptimal breath-holding, cardiac and respiratory motion artifacts, and limited signal-to-noise ratio (SNR). The short spiral readouts of this breath-held sequence limit SNR, which is why this study focused on the more anterior right coronary artery, as in most prior studies[9-13]. Increased BMI also likely contributed to reduced SNR, as obese patients (BMI ≥ 30, accounting for 24% of cohort) were less likely to have adequate image quality (39.2% vs. 81.7% for BMI < 30). Notably, patients with CAC≥400 were also less likely to have adequate MR image quality (52.5% vs. 70.5% for CAC < 400), suggesting that severe calcification (with low MR signal) limits coronary wall visualization. A higher proportion of our older cohort (78%) had evidence of coronary calcification compared to the MESA study (46%)[14].

Importantly, in those subjects with adequate image quality, quantitative measures had very good intra- and inter-observer agreement, which had only been shown previously in healthy volunteers[23,24]. Several approaches are likely needed in combination to improve image quality and SNR in order to make this a more robust and reliable technique. These include optimized multi-element surface coils, 3 D acquisition[10,23], free-breathing methods to allow longer acquisitions[26], radial *k*-space sampling[27], and higher magnetic field strength, such as 3T[28-31].

### Positive Arterial Remodeling

The compensatory enlargement of artery size with increased atherosclerosis to maintain lumen size has been termed positive arterial remodeling. Our finding of increased coronary vessel area and preserved lumen area with increased coronary wall thickness confirms, in an older population, the prior reports of positive remodeling by coronary wall CMR[10,14]. Importantly, while male gender and BMI can affect vessel size, these were not associated with increased wall thickness on multivariate analysis. This identification of positive remodeling is potentially valuable clinically, as it has been associated with an increased risk of plaque rupture by pathology[8].

### Diabetes and Subclinical Coronary Atherosclerosis

Among coronary risk factors, type 2 diabetes was the strongest predictor by multivariate analysis of increased coronary wall area and wall thickness by CMR in the present study. These results are consistent with autopsy and CT studies that have documented a higher prevalence of coronary atherosclerosis among patients with diabetes, even in the absence of symptoms or clinical evidence of coronary artery disease[32-34]. CMR of the RCA wall has been applied previously to a subset of 61 patients from a cohort of asymptomatic type 1 diabetics, with wall thickness found to be greater in patients who also had nephropathy[13]. In the MESA study, HDL and smoking history were associated with increased coronary wall thickness on univariate analysis, but no multivariate analysis data were provided[25]. The number of diabetics reported in MESA (n = 4) was likely too small for analysis of this association. Given the increasing incidence of type 2 diabetes, data on the prognostic utility of coronary wall CMR to identify diabetic patients at increased risk of progression to clinical coronary heart disease would clearly be valuable.

### Coronary Wall CMR and Coronary Artery Calcification

CAC measured by CT has been the most widely used direct noninvasive measure of coronary atherosclerosis[35] and is associated with the extent of coronary atherosclerosis by pathology and clinical events[33,36]. Black-blood CMR can assess coronary wall thickness, but does so over a more limited region. Thus, CAC by CT and coronary wall thickness by CMR provide different measures of coronary atherosclerosis. We found a significant association of RCA coronary wall thickness with both total CAC and CAC of the RCA, similar to the MESA study[14]. Further studies comparing coronary CMR to CAC, as well as CT angiography, are clearly needed.

### Study Limitations

There is a growing literature of coronary wall CMR studies, but limited validation with intravascular ultrasound, which cannot be justified in asymptomatic subjects. We imaged only a single region of the RCA, as in most prior studies, as the RCA is closer to the chest wall providing higher SNR and good reproducibility[10,23]. Atherosclerosis is a systemic disease that can cause a diffuse increase in coronary wall thickness, but more comprehensive imaging to also detect and characterize focal lesions would be desirable. The MESA study did include selected left main and left anterior descending artery locations, but ideally whole-heart coronary wall CMR methods are needed. Finally, the ultimate value of coronary wall CMR will be in predicting clinical events and guiding therapy.

## Conclusions

Noninvasive right coronary wall CMR was successfully performed in a community-based cohort of older subjects, demonstrating positive arterial remodeling and association with type 2 diabetes and coronary artery calcification. Coronary wall CMR warrants further study in the detection of subclinical coronary atherosclerosis and prediction of clinical cardiovascular events.

## Competing interests

MVM and DGN receive research support from GE Healthcare. MVM has been an advisor for Philips Healthcare and Kowa, Inc. MT has received honoraria from Philips Healthcare. GDR has been an advisor for TeraRecon, Inc. and Siemens Medical Solutions. AS is an employee of GE Healthcare.

## Authors' contributions

MT performed CMR studies, data analysis, and drafted the manuscript. PKN performed CMR studies and data analysis. GDR participated in the study design and supervised the coronary CT exams and data analysis. CHM, AS, and DGN helped develop and implement the CMR methods. SE contributed to the data analysis. BC contributed to the CMR image analysis methods. CI, ASG, MAH, and SPF participated in the study design and contributed to data analysis methods. MVM participated in the study design, patient studies, and data analysis. All authors have participated in critical review of the manuscript and have read and approved the final version.
